# Genetic Susceptibility to Leprosy—From Classic Immune-Related Candidate Genes to Hypothesis-Free, Whole Genome Approaches

**DOI:** 10.3389/fimmu.2018.01674

**Published:** 2018-07-20

**Authors:** Geison Cambri, Marcelo Távora Mira

**Affiliations:** Graduate Program in Health Sciences, School of Medicine, Pontifícia Universidade Católica do Paraná, Curitiba, Paraná, Brazil

**Keywords:** leprosy, genetics, association studies, *PARK2*, next-generation sequencing, disease modeling

## Abstract

Genetics plays a crucial role in controlling susceptibility to infectious diseases by modulating the interplay between humans and pathogens. This is particularly evident in leprosy, since the etiological agent, *Mycobacterium leprae*, displays semiclonal characteristics not compatible with the wide spectrum of disease phenotypes. Over the past decades, genetic studies have unraveled several gene variants as risk factors for leprosy *per se*, disease clinical forms and the occurrence of leprosy reactions. As expected, several of these genes are immune-related; yet, hypothesis-free approaches have led to genes not classically linked to immune response. The *PARK2*, originally described as a Parkinson’s disease gene, illustrates the case: Parkin—the protein coded by *PARK2*—was defined as an important player regulating innate and adaptive immune responses only years after its description as a leprosy susceptibility gene. Interestingly, even with the use of powerful hypothesis-free study designs such as genome-wide association studies, most of the major gene effect controlling leprosy susceptibility remains elusive. One hypothesis to explain this “hidden heritability” is that rare variants not captured by classic association studies are of critical importance. To address this question, massively parallel sequencing of large segments of the human genome—even whole exomes/genomes—is an alternative to properly identify rare, disease-causing mutations. These mutations may then be investigated through sophisticated approaches such as cell reprogramming and genome editing applied to create *in vitro* models for functional leprosy studies.

## Introduction

Infectious diseases are essentially caused by pathogens capable to transpose the immunological barrier and colonize the host organism. Exposure to an infectious agent is necessary but not sufficient to determine disease; exposed organisms need to be naturally susceptible and even then, clinical disease outcomes often display marked interindividual variation (1). The explanation for such variability can be addressed to different reasons, including environmental factors, divergence in virulence of pathogen strains and particularly, to the complex interplay between host and pathogens. A remarkable demonstration of this variability was observed in the Lübeck disaster occurred in the late 1920s: 251 neonates were accidentally infected by virulent *Mycobacterium tuberculosis* contaminating a batch of Bacille Calmette-Guérin (BCG) vaccine. Twenty-three infants (9.2%) did not show any clinical signs of tuberculosis and the mortality rate was 29%; 68% of neonates who presented clinical disease spontaneously progressed to cure (2).

Robust evidence that the host–pathogen interplay is largely influenced by the genetic make-up of the host has been brilliantly demonstrated in an adoptee study: predisposition to infectious disease was predominantly inherited, in an interesting contrast with cancer that was found to be much more dependent of non-genetic factors (3). Innate predisposition to infection seems to be particularly crucial for leprosy: it is estimated that only a small fraction (from 5 to 12%) of individuals exposed to *Mycobacterium leprae* are successfully infected (4, 5). Although leprosy is treatable by an efficient multidrug regimen available for free around the world, latest reports from 143 countries show 214,783 new cases, with India (63.08%), Brazil (11.74%), and Indonesia (7.83%) presenting the highest percentage of registered cases (6). Patients display a wide spectrum of clinical phenotypes that are related to individual differences in immune response and distributes between two poles: in one extreme, tuberculoid patients presents a strong cellular (Th1) immune response with increased production of pro-inflammatory cytokines such as interleukin-2 and interferon-γ and a low or inexistent bacillary load in lesions; on the other extreme of the spectrum, lepromatous leprosy is characterized by a predominantly antibody-based (Th2) immune response with predominant expression of interleukin-10 and interleukin-4 and a high number of *M. leprae* in skin smears. Borderline disease displays a gradient of immune features depending on the proximity to one of the poles (7, 8). During the course of the disease, treatment or even after cure, up to 50% of patients develop one of the two types of an aggressive, sudden inflammatory response known as leprosy reaction, the major cause of permanent neural damage with consequent disabilities today (9, 10).

**Figure 1:**
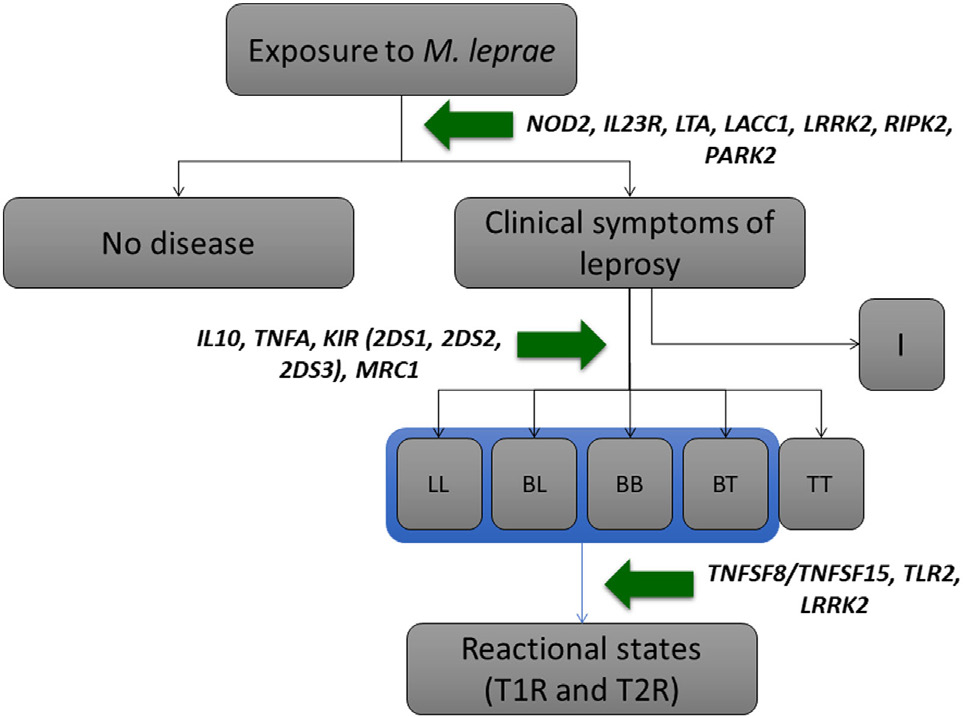
Pathogenesis of leprosy with selected genes impacting on its phenotypes. Adapted from Mira (12) and Sauer et al. (13). For a more detailed list of genes, please refer to Table 1. Abbreviations: I, indeterminate; LL, lepromatous leprosy; BL, borderline-lepromatous; BB, borderline–borderline; BT, borderline-tuberculoid; TT, tuberculoid–tuberculoid; T1R, type-1 reaction; T2R, type-2 reaction.

The *M. leprae* is an acid-fast, Gram-positive bacillus incapable of growing in axenic media, thus strongly dependent on the host cellular environment. The bacterium presents a reduced genome and semi-clonal characteristics across strains distributed worldwide (11), reinforcing the impact of host genetics over control of disease *per se*, its clinical forms, and the occurrence of leprosy reactions. Decades of extensive research positions host genetics as a major player controlling susceptibility to leprosy (12, 13). Early evidence comes from genetic descriptive, DNA-free studies: leprosy occurrence displays strong familial aggregation (14) and concordance of infection is higher in monozygotic (59.7%) as compared to dizygotic twins (20%) (15). Several complex segregation analysis (CSA) consistently revealed the presence of a major gene effect controlling susceptibility to leprosy *per se* in different population samples of distinct genetic backgrounds, although no consensus on the exact model of inheritance has been achieved (16, 17). Later, hypothesis-free genome-wide linkage scans have identified chromosomal regions such as 10p13, 6q25-27, and 6p21 as positional candidates to harbor leprosy susceptibility genes (18, 19), and the first Genome-Wide Association Study (GWAS) on leprosy has been performed using a large Han Chinese sample set: a total of 491,883 single nucleotide polymorphisms (SNPs) spanned over the genome were first genotyped in 706 patients and 1,225 controls; the 93 markers associated with the smallest *p*-values were later tested for replication in two additional independent population samples (20). Combined, these molecular strategies have led to the description of a multitude of genes associated to leprosy (Figure 1; Table 1), several of them participating in host immune response and/or bacterial routes of infection and evasion from the immunological barrier.

**Table 1:** Leprosy-associated genes with functional evidence or replicated status.

Gene	Name	Identification strategy	Population sample (Reference)	Gene function/pathway
*CARD9*	Caspase recruitment domain family member 9	GWAS—protein-coding variants	Chinese case-control (21)	Regulatory function in cell apoptosis and induction of NF-kB

*FLG*	Filaggrin	GWAS—protein-coding variants	Chinese case-control (21)	The structural component of the epidermis

*HIF1A*	Hypoxia-inducible factor 1 alpha subunit	GWAS—protein-coding variants	Chinese case-control (22)	Regulator of cellular and systemic homeostatic response to hypoxia; inflammation, autophagy, and immune reactions

*HLA-C*	Major histocompatibility complex (MHC), class-I. Human leukocyte antigen (HLA)	Association scan of the HLA *locus*	Vietnamese family-based (23) Indian case-control (23)	Immune recognition and antigen presentation

*HLA-DR-DQ*	MHC, class-II. HLA	GWAS	Chinese case-control (20)	Immune recognition and antigen presentation
		Association scan	Indian case-control (24)	
		Candidate gene analysis	Vietnamese family-based (25)	

*IL10*	Interleukin 10	Candidate gene analysis	Brazilian case-control (26)	Immunoregulation; downregulates Th1 response and induces B-cell survival, proliferation, and antibody production
			Brazilian case-control (27)	
			Indian case-control (28)	
			Brazilian family-based; meta-analysis (29)	

*IL12B*	Interleukin 12B	GWAS	Chinese case-control (30)	Activator of NK and T-cells. Inducer of Th1 immune response

*IL18RAP/IL18R1*	Interleukin 18 receptor accessory protein/interleukin 18 receptor 1	GWAS	Chinese case-control (30)	The receptor of IL18, a proinflammatory cytokine that induces cell-mediated immune response

*IL23R*	Interleukin 23 receptor	GWAS—protein-coding variants	Chinese case-control (21)	Binds to IL23 activating NK and T-cells; pro-inflammatory receptor
		GWAS	Chinese case-control (31)	

*IL27*	Interleukin 27	GWAS—protein-coding variants	Chinese case-control (21)	Modulator of T-cell differentiation

*KIR* (*2DS1, 2DS2, 2DS3*)	Killer immunoglobulin-like receptor (KIR)	Candidate gene analysis	Brazilian case-control (32)	Regulatory molecules of NK cells surface; mediates NK reactivity against target cells; depending on HLA-I ligands
			Brazilian case-control (33)	

*LACC1—CCDC122*	Laccase domain containing—coiled-coil domain containing 122	GWAS	Chinese case-control (20)	LACC1 is involved in fatty-acid oxidation with inflammasome activation, ROS production, and modulation of bactericidal activity of macrophages. CCDC122 function is presently unknown
			Chinese case-control (34)	
		Candidate gene analysis	Brazilian family-based (35)	
			Brazilian case-control (35)	
			Vietnamese family-based (25)	
		GWAS—protein-coding variants	Chinese case-control (22)	

*LRRK2*	Leucine rich repeat kinase 2/Dardarin	GWAS	Chinese case-control (20)	Regulation of autophagy, inflammasome activity, and production of ROS and inflammatory cytokines
		Candidate gene analysis	Indian case-control (36)	
			Chinese case-control (37)	
			Vietnamese family-based (38)	

*LTA*	Lymphotoxin-α	Genome-wide linkage analysis	2 Vietnamese family-based (39)	Pro-inflammatory cytokine, it mediates inflammatory response
			Brazilian case-control (39)	
			Indian case-control (39)	

*MRC1*	Mannose receptor C-type 1	Candidate gene analysis	Vietnamese family-based (40)	Membrane receptor that mediates carbohydrate recognition
			Brazilian case-control (40)	

*NCKIPSD*	NCK interacting protein with SH3 domain	GWAS—protein-coding variants	Chinese case-control (21)	Signal transduction; regulation of cytoskeleton

*NOD2*	Nucleotide-binding oligomerization domain containing 2	GWAS	Chinese case-control (20)	Recognition of LPS bacterial structure and activation of NF-kB
		Candidate gene analysis	Brazilian family-based (35)	
			Brazilian case-control (35)	
			Vietnamese family-based (25)	

*PARK2*	Parkin RBR E3 ubiquitin protein ligase	Genome-wide linkage analysis	Vietnamese family-based (41)	E3 ubiquitin-protein ligase with a role on proteasome function, mitophagy, intracellular bacterial clearance, and mitochondrial antigen presentation
			Brazilian case-control (41)	
		Candidate gene analysis	2 Indian case-control (42)	
			Vietnamese family-based (43)	

*RAB32*	RAB32, member RAS oncogene family	GWAS—protein-coding variants	Chinese case-control (21)	Protein metabolism, vesicle-mediated transport and autophagy
		GWAS	Chinese case-control (31)	

*RIPK2*	Receptor-interacting serine/threonine kinase 2	GWAS	Chinese case-control (20)	Signaling, innate and adaptive immune response; NF-kB inducer
			Chinese case-control (34)	
		Candidate gene analysis	Vietnamese family-based (25)	

*SLC29A3*	Solute carrier family 29 member 3	GWAS—protein-coding variants	Chinese case-control (21)	Nucleoside transporter

*TLR1*	Toll-like receptor 1	GWAS	Indian case-control (24)	Pathogen recognition and activation of innate immunity
		Candidate gene analysis	Brazilian case-control (44)	
			Brazilian family-based (44)	

*TLR2*	Toll-like receptor 2	Candidate gene analysis	Ethiopian case-control (45)	Pathogen recognition and activation of innate immunity

*TNFA*	Tumor necrosis factor alfa	Candidate-gene analysis	Brazilian family-based and case-control; meta-analysis (46)	Pro-inflammatory cytokine

*TNFSF8/TNFSF15*	Tumor necrosis factor (Ligand) Superfamily, Member 8/Member 15	GWAS	Chinese case-control (20)	Pro-inflammatory cytokine
		Candidate gene analysis	Vietnamese family-based (47)	
			Brazilian case-control (47)	

*TYK2*	Tyrosine kinase 2	GWAS—protein-coding variants	Chinese case-control (21)	Cytokine modulator, interferon signaling pathway

*GWAS, Genome-wide association study; ROS, reactive oxygen species; LPS, lipopolysaccharides; NK, natural killer*.

A natural functional and positional candidate genomic region has been the major histocompatibility complex (MHC)/human leukocyte antigen (HLA) located in the highly polymorphic 4 Mb interval at chromosome 6p21. The complex is essential for recognition, processing, and presentation of antigens during immune response. Genes located in all three MHC/HLA classes have been exhaustively studied in leprosy and haplotypes have been associated with both susceptibility and protection against the disease in distinct populations (13, 48). Killer immunoglobin-like receptors genes—*KIR2DS1, 2DS2*, and *3DS1*—and their HLA ligands were associated with leprosy in a Brazilian population (32, 33) and *HLA-C*, a classical ligand for KIRs, was observed as a risk factor for leprosy in Vietnamese family based and Indian case-control populations (23). Genetic variants in the class-II *HLA-DR–DQ* locus have been consistently associated with protection against leprosy (20, 24). In the MHC class-III region, linkage disequilibrium mapping of the 6p21 region identified the low-producing A allele of the variant + 80 of Lymphotoxin-α (*LTA* + *80*) as a risk factor for infection: association was reported in a Vietnamese, family-based sample and validated in a Brazilian case-control sample (39).

Receptors for pathogen-associated molecular patterns, classic molecules of the innate immune response, have been also consistently associated with leprosy. The non-synonymous single-nucleotide polymorphism G396S located at the Mannose Receptor C-type lectin (*MRC1*) gene located in region 10p13 was described as a risk factor for leprosy susceptibility in different populations (40). Polymorphisms in the toll-like receptor (TLR) family were repeatedly associated with leprosy and its phenotypes. Amino acid substitutions N248S and I602S in the *TLR1* gene have been associated with susceptibility (44) and protection (24) against leprosy, respectively. SNP markers 597 C/T (rs3804099) and a 280 bp-length microsatellite of *TLR2* have been associated with protection and increased risk of leprosy reactions, respectively (45) Another sensing molecule consistently associated with leprosy is the nucleotide-binding oligomerization domain 2 (NOD2), a cytoplasmic receptor responsible for recognizing intracellular pathogens *via* their peptidoglycan components of the bacterial cell wall. *NOD2* involvement in leprosy was first identified in a GWAS (20) and later replicated (35); in addition, association of *NOD2* variants with leprosy reaction has been detected (49). Functionally analysis has demonstrated that a structurally unique muramyl dipeptide of *M. leprae* is recognized by NOD2, triggering expression of interleukin-32 and monocytes differentiation into dendritic cells (50).

Cytokines regulating the Th1/Th2 immune responses have also been described associated with leprosy phenotypes. *TNFA* and *IL10* genetic variants are classic risk factors for leprosy (29, 46); gene products TNF-α and IL-10 are major signature cytokines for the tuberculoid and lepromatous pole, respectively (51). More recently, GWAS have suggested a role in leprosy susceptibility control of *IL12B, IL27*, and pro-inflammatory receptors *IL23R* and *IL18RAP/IL18R1* that regulates the adaptive immune response (21, 30). Functional assays indicate regulation of IL10 expression by IL27, inhibiting host defense through IFN-γ-induced antimicrobial activity (52).

A more comprehensive analysis of leprosy genetic studies reveals a complex network of interactions among associated genes. This is well exemplified by *LRRK2*, initially identified in the first leprosy GWAS (20) and later replicated in an Indian population (36); *LRRK2* participates in the control of autophagy with involvement of the small GTPase RAB32 (53), which gene is associated with leprosy in two unrelated GWAS (21, 31). Interestingly, LRRK2 is also correlated to bacterial survival and co-localization as observed in RAW 264.7 cells infected by *Salmonella typhimurium* (54); although, the increase of LRRK2-kinase activity increases *M. tuberculosis* survival through reduction of phagosome maturation (55). Recently, *LRRK2* has been associated with leprosy type-1-reaction, a pathological inflammatory response event (38). Finally, *LRRK2* is a negative regulator of inflammasome activation (56, 57) and an inducer of ROS production (54, 57), two known mechanisms of immune defense against bacterial infections also modified by variants of *LACC1*, a gene consistently associated with leprosy (20, 25, 34, 35). Recently, the *LACC1* contribution to leprosy risk has been reinforced: a GWAS-based analysis focusing on functional variants detected association between leprosy and a *LACC1* missense variant (rs3764147, c.760A > G, p.Ile254Val) (22).

Several other genes enrolled in immune-response pathways have been associated to leprosy and its phenotypes; however, a full description of these studies goes beyond the scope of this paper, for more detailed data, please refer to Table 1 and Ref. (12, 13, 58, 59).

An intriguing aspect revealed by leprosy hypothesis-free genetic studies is the often identification of genes not classically related to immune response pathways—genetics studies on leprosy have contributed to the description of unsuspected immune-related roles for these genes; Parkin, the protein coded by *PARK2*, illustrates the case.

## The PARK2/Parkin Case

The *PARK2* gene was originally described in 1998 as a result of an investigation of microdeletions in patients carrying autosomal recessive juvenile parkinsonism (AR-JP): the authors isolated a 2,960 bp DNA sequence containing an open reading frame coding for a 465 amino acid protein. Characterization of the sequence by alignment and screening of DNA libraries led to the discovery of a ubiquitin-like protein, named Parkin due to its impact on Parkinson disease (60). Two years later, the *PARK2* gene product was defined as a ubiquitin-protein ligase and its loss of function reputed as causal of AR-JP (61).

First evidence of a role for *PARK2* in leprosy control came from a genome-wide linkage analysis. Genotyping of 388 microsatellite markers covering the whole genome (10 cM interval) was conducted in 86 Vietnamese families displaying 205 affect siblings; 11 chromosome regions were initially linked to leprosy. In a second-round of genotyping, all 11 regions were saturated with additional 89 markers and results evidenced strong co-segregation of the 6q25-q27 region and leprosy (maximum likelihood binomial LOD score 4.31; *P* = 5 × 10^−6^) (19). In a follow-up study, association fine mapping of the 6q25-27 genomic region using 208 independent simplex Vietnamese families lead to the discovery of SNPs clustered in the shared promoter region of *PARK2* and *PACRG* genes, associated with increased risk of leprosy in two ethnically independent population, Vietnamese and Brazilian. Linkage-disequilibrium analysis evidenced two tag-SNPs—common allele “T” from PARK2_e01(-2599) and rare allele “C” from rs1040079—capturing the complete association information (41). Interestingly, *PARK2*, a non-immune related gene by the time of the study, was the first gene described and validated as having an impact on susceptibility to leprosy by a hypothesis-free, positional cloning strategy.

*PARK2* association with leprosy was further replicated in an Indian population; however, association signal did not pass a conservative Bonferroni correction for multiple testing (62). Polymorphisms in *PARK2*/*PACRG* co-regulatory region were also found associated with leprosy risk in Croatian (63) and two unrelated Indians population samples (42). Moreover, the association was confirmed in independent Vietnamese and Indian samples with a remarkable contribution of age-at-diagnosis to the association signal (43). *PARK2’s* impact over susceptibility to infection was also demonstrated by association of the T allele-2599 to typhi and paratyphoid fever, diseases caused by *Salmonella*, an intracellular pathogen (64).

Parkin is an E3 ubiquitin-ligase involved in the proteasome pathway, in particular, the autophagy cellular mechanism of turnover of damaged biomolecules (lipids and proteins) and organelles. Parkin targets are marked and delivered to autophagosomes that are fused with lysosomes and consequently degraded. Of particular importance is the role of Parkin in the mitophagy pathway of mitochondrial recycling: along with PTEN-induced putative kinase protein 1 (PINK1), a mitochondrial kinase, Parkin modulates mitochondrial quality control by mediating the ubiquitination of mitochondrial proteins when the organelle is depolarized (65).

Autophagy has been described as an important defense mechanism aiming to destroy intracellular pathogens, an innate immune response process named xenophagy (66). Through this mechanism, invading microbes are labeled with ubiquitin and adaptor proteins (e.g., p62, NDP52, and optineurin) to be presented to autophagy protein LC3 and initialize assembly of the autophagosome (67). Bacterial degradation by xenophagy has been described against mycobacteria, including *M. tuberculosis* (68). More recently, Parkin has been described to participate in this pathway mediating resistance against *M. tuberculosis* and *Salmonella enterica* serovar Typhi. Parkin is essential for colocalization of ubiquitin along phagosomes markers within *M. tuberculosis*; murine bone-marrow-derived-macrophages bearing double knockouts for *PARK2* are more susceptible to *M. tuberculosis* or *S. enterica* growth and present a decrease in survival rate after infection (69). Parkin role in the clearance of intracellular bacteria is corroborated by functional assays performed using dendritic cells infected by Chlamydia: autophagosome degradation of chlamydial infections and MHC-I antigen presentation are increased in presence of Parkin (70).

The influence of Parkin in T-cell stimulation has been also demonstrated in the mitochondrial antigen presentation (MitAP) pathway, based on the generation and trafficking of mitochondrial-derived vesicles (MDV) and mediated by Parkin and PINK1. Under stress, Sorting Nexin-9 (Snx9) and the GTPase Rab9 are recruited to mitochondria and triggers MDV formation; Parkin modulates this process by regulating the level of Snx9 in the cytosol in a proteasome-dependent manner, consequently repressing MitAP in antigen-presenting cells and impacting over immune tolerance (71). It is worth to note that MitAP is not mediated by mitophagy; also, Parkin has an effect upon the production of interleukin-6 and monocyte chemoattractant protein-1 (MCP-1/*CCL2*) (72), suggesting an impact of Parkin in multiple pathways related to immunity. Interestingly, this impact seems to be conserved among species since impairment of autophagic activity and lifespan after infection is observed in *Drosophila melanogaster* in the absence of *PARK2* expression (69, 73).

In summary, genetic and functional studies have provided strong evidence of *PARK2* as a key player in the pathogenesis of leprosy and other infectious diseases. However, these exciting findings are not enough to explain the strong genetic effect observed and estimated through CSA and twin studies—causal variants with high penetrance, able to explain the major gene effect, are yet to be evidenced. The strategies presented next might be powerful to contribute to the advance of the complete dissection of the molecular basis of susceptibility to infection.

## Strategies and Future Perspectives

A main genetic assumption underlying classic genetic epidemiology studies is that common diseases are caused by common variants [i.e., nucleotide changes with Minor Allelic Frequency (MAF) > 1%]. Thus, genetic study designs, including GWAS, have been focusing on identifying these common variants and several have been associated to leprosy, some with consistent replication/validation across populations of distinct genetic backgrounds. This positive scenario led to the expectation that these powerful studies would reveal most—if not all—of the genetic variation impacting on susceptibility to common diseases in general and leprosy in particular. However, the picture that emerges today is distinct: GWAS have been revealing a large number of common variants associated with complex traits with very low odds ratios, and the combined effects explain ~5% or less of genetic variance to a given trait (74). These observations led to the term “missing heritability,” which can be at least partially explained by rare variants (MAF < 1%) with larger effects on phenotype or variants other than SNPs, such as copy number variants, both poorly represented in typical genotyping arrays (75). To address this hypothesis, massive deep sequencing technology has been proved to be a powerful tool. In recent years, advances in DNA sequencing chemistry and platforms have allowed an enormous improvement in data generation with a reduced cost (76). Therefore, human whole-genome sequencing (WGS) or whole-exome sequencing (WES) have become feasible and these approaches, especially WES has been proven effective to the identification of genes underlying several Mendelian diseases (77–79). Alternative designs might be used to reduce costs, improve power of detection, and increase individual sample sizes for sequencing; an insightful approach for leprosy might be to submit genes consistently associated with the disease to deep sequencing and search for new/rare variants as causal candidates. Moreover, exons can be preferentially targeted, as variants that cause amino acid change are more likely to have an impact on the phenotypes (80). This rationale was recently used to identify a common and a rare missense functional variant of *LACC1* and *HIF1A*, respectively, as risk factors for leprosy, using WES and targeted second-generation sequencing (22). Another powerful approach has been the use of WGS/WES on the investigation of families or patients displaying extreme or atypical phenotypes; for example, individuals who do not present clinical disease albeit being exposed to an infectious microorganism, as it has been demonstrated for HIV (81, 82). As variants will be likely enriched in such cases, the discovery of causal mutations could be performed in smaller samples size (77).

A natural step further following genetic variant discovery is functional testing. Advances in genome edition technology and cell reprogramming have been allowing isogenic models ideal for functional studies on complex diseases. Such approaches have been proven useful to study neurological diseases such as Huntington’s (83), Parkinson’s (84), and Alzheimer’s disease (85). Genomic variants can be edited by CRISPR/Cas9 in the presence of a donor DNA harboring the nucleotide change; after DNA cleavage by Cas9 nuclease, the homology-directed repair machinery is activated and the donor-DNA is inserted (86), creating a feasible strategy to perform *in vitro* disease modeling with isogenic controls. In a potentially powerful combination, genome editing strategies could be applied to modify induced pluripotent stem cells (iPSC) (87) that could be differentiated into cell types, for example, targets of a specific pathogen. Although, Cas9 edition system might display off-targets, tools to reduce off-targets mutations have been developed, such as the use of Cas9 in a ribonucleoprotein complex (88) and nickases-Cas9 (89), which cleaves a single strand of DNA, thus a complementary pair of anti-sense gRNAs is necessary to induce mutation (90).

## Conclusion

Genetics studies have significantly contributed to the understanding of the molecular basis of leprosy susceptibility and the pathophysiology of the disease. Interestingly, genome-wide, hypothesis-free studies led to the discovery of unsuspected immune-related genes such as *PARK2* in the past and, more recently, *LACC1* (22, 57, 91). Yet, the impact of rare variants upon disease mechanisms is largely unknown and causal variants that could explain the major gene effects are yet to be described. Advances in genome sequencing technology and functional studies approaches might contribute substantially to further advances in leprosy and other infectious/common diseases.

## Author Contributions

GC designed the manuscript and performed major writing. MM contributed to the writing and provided senior supervision.

## Conflict of Interest Statement

The authors declare no conflict of interest; the research was conducted in the absence of any commercial or financial relationships that could be construed as a potential conflict of interest.
